# Analysis and effect of conventional flasks in shaking culture of *Escherichia coli*

**DOI:** 10.1186/s13568-020-01013-7

**Published:** 2020-04-19

**Authors:** Masato Takahashi, Hideki Aoyagi

**Affiliations:** Faculty of Life and Environmental Sciences, University of Tsukuba, Tsukuba, Ibaraki 305-8572 Japan

**Keywords:** Aerobic culture, Carbon dioxide, *Escherichia coli*, Headspace, Monitoring device, Shake-flask culture

## Abstract

The circulation direct monitoring and sampling system (CDMSS) is used as a monitoring device for CO_2_ and O_2_ concentrations of bypass type in shake-culture flask. The CDMSS could measure *k*_L_a, an index for evaluating the performance of aerobic culture incubators, and *k*_G_, an indicator of the degree of CO_2_ ventilation in the flask gas phase. We observed that cylindrical flasks provided a different culture environment, yielded a much higher *k*_G_ than the Erlenmeyer and Sakaguchi flasks, and yielded *k*_L_a equivalent to that by Erlenmeyer flask by setting the ring-type baffle appropriately. Baffled cylindrical flask used for *Escherichia coli* K12 IFO3301 shake culture maintained lower CO_2_ concentrations in the headspace than conventional flasks; therefore, CO_2_ accumulation in the culture broth could be suppressed. Cell growth in baffled cylindrical flask (with *k*_L_a equivalent to that of the Erlenmeyer flask) was about 1.3 and 1.4 times that in the Erlenmeyer and Sakaguchi flasks, respectively. This study focused on the batch culture at the flask scale and designed the headspace environment with low CO_2_ accumulation. Therefore, we conclude that redesign of flasks based on *k*_L_a and *k*_G_ may contribute to a wide range of fields employing microorganism culture.

## Introduction

In 1932, shake-flask culture was developed for submerged culture of fungi to overcome the biomass yield limitations of surface culture (Kluyver et al. 1933). Erlenmeyer flask is now widely used to culture microorganisms and plant and animal cells (Weirether et al. 1968; Klöckner and Büchs 2012; Platas et al. 2013; Reynoso-Cereceda et al. 2016; Eibl et al. 2018). Shake-flask culture of microorganisms is aerobic and aims to provide sufficient stirring and oxygen to the culture broth and enables microorganisms to be batch cultured in parallel at low cost. Therefore, shake-flask culture is frequently used to screen secondary metabolites and to optimise culture conditions for microorganisms in the initial steps of bioprocess development. However, unlike in the case of agitated bioreactors (such as jar fermenters), the various culture conditions in shake flasks generally cannot be monitored without dedicated devices.

Monitoring technology in shake flasks has been becoming popular. Gaseous monitoring devices are remarkable because accurate measurement with conventional sampling methods, which include the interruption of shake culture and the transporting to a clean bench, the opening of culture plug at clean bench, are difficult to achieve due to Henry’s law (Takahashi et al. 2017; Takahashi and Aoyagi 2018a). The methods of monitoring gas concentration in shake-flask culture are characterised by the measuring site, the principle of measurement equipment, and the measurement style (Takahashi and Aoyagi 2018b). The standard direct device is the Respiration Activity Monitoring System (RAMOS), which can monitor the flask gas phase (Anderlei et al. 2004). To the best of our knowledge, the Circulation Direct Monitoring and Sampling System (CDMSS) is the first bypass device to be developed that can monitor the behaviour of CO_2_ and O_2_ in the gas–liquid phases and obtain a sample without interrupting the shaking of the culture (Takahashi et al. 2017). A device that combines the direct type and the bypass type has also been recently reported (Schulte et al. 2018). Most monitoring devices for shake-flask cultures remain only an implementation to Erlenmeyer flask (Anderlei et al. 2004; Ge and Rao 2012; Takahashi et al. 2017). Alternatively, several studies have focused on the shake culture compatibility between an Erlenmeyer flask and microplate, based on the same measurement principle of RAMOS (Wewetzer et al. 2015). On the contrary, in shake-flask culture, not only Erlenmeyer flasks, but also Sakaguchi flasks, are frequently used in reciprocal shaking (Shiota and Sakaguchi 1950; Omura et al. 1977; Hirasawa et al. 2006; Nojiri et al. 2015; Matsuda et al. 2017). There are few reports on monitoring of culture broth in Sakaguchi flask, and only one report on the headspace of Sakaguchi flask, which identified a concentration gradient in the vertical direction of the gas phase (Takahashi and Aoyagi 2018c). There is no research yet on monitoring and comparison of the CO_2_ and O_2_ concentrations in the headspace and culture broth in shake culture in conventional flasks, such as Erlenmeyer and Sakaguchi flasks. There are not sufficient studies on the relationship between flask shape and CO_2_ and O_2_ in shake-culture flasks of microorganisms.

Our study examined the behaviour of *Escherichia coli* K12 IFO3301 shake culture in various flasks by using circulation direct monitoring and sampling system (CDMSS). CDMSS was also used to calculate the total oxygen transfer capacity coefficient (*k*_L_*a*), which is an indicator of the capacity to supply oxygen from the headspace to the liquid via the gas–liquid interface, and *k*_G_, which is an indicator of CO_2_ ventilation from the headspace through the breathable stopper into the atmosphere, in Erlenmeyer and Sakaguchi flasks. Based on these findings, we utilised and evaluated the flasks of cylindrical shape with CO_2_ ventilation capacity in the headspace, which cannot be obtained in Erlenmeyer and Sakaguchi flasks.

## Materials and methods

### Microorganisms, medium, and inoculum preparation

*Escherichia coli* K12 IFO3301 was selected as the experimental organism. The LB medium (pH 7.0) used to culture *E. coli* K12 IFO3301 consisted of: (in g/L) tryptone, 10; yeast extract, 5; and NaCl, 5. A loop-full of *E. coli* K12 IFO3301 slant culture was inoculated into a 500-mL Erlenmeyer flask containing 100 mL of LB medium. The sample was then cultured at 30 °C on a rotary shaker with 70 mm shaking diameter at 200 rpm for 7.5 h. Glycerol stocks were prepared by adding the culture medium to glycerol (final glycerol concentration: 20% [v/v]) and stored at − 80 °C.

### Culture conditions

Erlenmeyer, Sakaguchi, cylindrical, and baffled cylindrical flasks were selected for the study. All flasks were 500-mL in size. A detachable, O-ring shaped baffle was selected for constructing the baffled cylindrical flask (6 cm from the bottom). One mL each of glycerol stock was inoculated into a 500-mL Erlenmeyer flask and a 500-mL cylindrical flask containing 100 mL of LB medium, respectively, and cultured at 30 °C on a rotary shaker with 70 mm shaking diameter at 200 rpm. In the case of the 500-mL Sakaguchi flask containing 100 mL of LB medium, 1 mL of glycerol stock was inoculated and cultured at 30 °C on a reciprocating shaker with 70 mm shaking diameter at 120 strokes/min. Lastly, 50 mL of LB medium was added to the 500-mL baffled cylindrical flask and then 0.5 mL of glycerol stock was inoculated, cultured in the same way as Erlenmeyer flask and cylindrical flask. The shaking conditions of the Erlenmeyer flask and the Sakaguchi flask were those of the most frequently used rotary type and reciprocating type, respectively, which are standard culture conditions of *E. coli*.

### Measurement of *k*_L_a

Dissolved oxygen concentration was measured with the CDMSS using the sulfite oxidation method, and the total O_2_ transfer capacity coefficient (*k*_L_*a*) was calculated as follows:

1 $$- \ln \frac{{C_{\hbox{max} } - C}}{{C_{\hbox{max} } - C_{0} }} = k_{L} a \cdot t$$

where *t* is time (s), C is the measurement value of dissolved oxygen concentration, C_0_ is the dissolved oxygen concentration at *t* = 0 (mg/L), and C_max_ is the maximum dissolved oxygen concentration under the above-mentioned conditions. The maximum dissolved oxygen concentration was determined just before the measurements were taken for all conditions. When the decision coefficient value (termed *R*^2^) of the approximate expression of Eq. 1 exceeded 0.90, the slope of the graph was set as *k*_L_*a*.

Preliminary experiments confirmed that the O_2_ concentration in the headspace of all the flasks decreased during measurement. The headspace was continuously suctioned (200 mL/min) by using the CDMSS and the suctioned gas was exhausted without circulation to the headspace to prevent a decrease in the O_2_ concentration. In all the experiments for *k*_L_*a* determination, dissolved oxygen was measured while confirming that the O_2_ concentration in the headspace was equivalent to that in the atmosphere. The shaking conditions were constant at 200 rpm for the Erlenmeyer and cylindrical flasks and 120 strokes/min for the Sakaguchi flask. The working volume was set to 100 or 50 mL, and the *k*_L_*a* for each condition was calculated.

### Measurement of *k*_G_

In this study, it was observed that the CO_2_ concentration in the headspace of shake culture in the Erlenmeyer and Sakaguchi flasks was very high compared with that in the atmosphere. Consequently, we measured CO_2_ concentration in the headspace of flasks with shaking in a non-steady state by using the CDMSS. *k*_G_, an indicator of CO_2_ ventilation capacity, was calculated as follows:

2 $$- \ln \frac{{C_{\hbox{min} }^{{\prime }} - C{\prime }}}{{C_{\hbox{min} }^{{\prime }} - C_{0}^{{\prime }} }} = k_{\text{G}} \cdot t$$

where *t* is time (s), ${\text{C}}_{0}^{{\prime }}$ is the dissolved CO_2_ concentration at *t* = 0 (mg/L), and ${\text{C}}_{\hbox{min} }^{{\prime }}$ is the minimum CO_2_ concentration in fresh air. The minimum CO_2_ concentration, a very important factor for Eq. 2, was determined just before measurements were taken under all conditions.

Under all conditions, we obtained data until CO_2_ concentration decreased from 3.5 to 0.5%. When the *R*^2^ value of the approximate expression of Eq. 2 exceeded 0.990, the slope of the graph was set as *k*_G_. CO_2_ was added to the headspace of each flask via breathable culture stoppers until its level reached at least 5%. Shaking was initiated and after the CO_2_ concentration in the headspace had decreased to 3.5%, headspace CO_2_ concentration was measured every 20 s.

### Measurement of culture factors

The UOD_660_ (unit optical density at 660 nm) and pH of the culture broth were measured, which was sampled without interrupted shaking by CDMSS, using V-570 spectrophotometer (JASCO, Tokyo, Japan) and pH meter (HORIBA, Kyoto, Japan), respectively. To ensure minimal decrease in the volume of the culture broth owing to sampling from the same flask, the total sampling volume was maintained at < 10% of the total amount of the initial culture medium. All measurements were performed in duplicates.

### Monitoring of CO_2_ and O_2_ concentration in shake-culture flasks

**Fig. 1:**
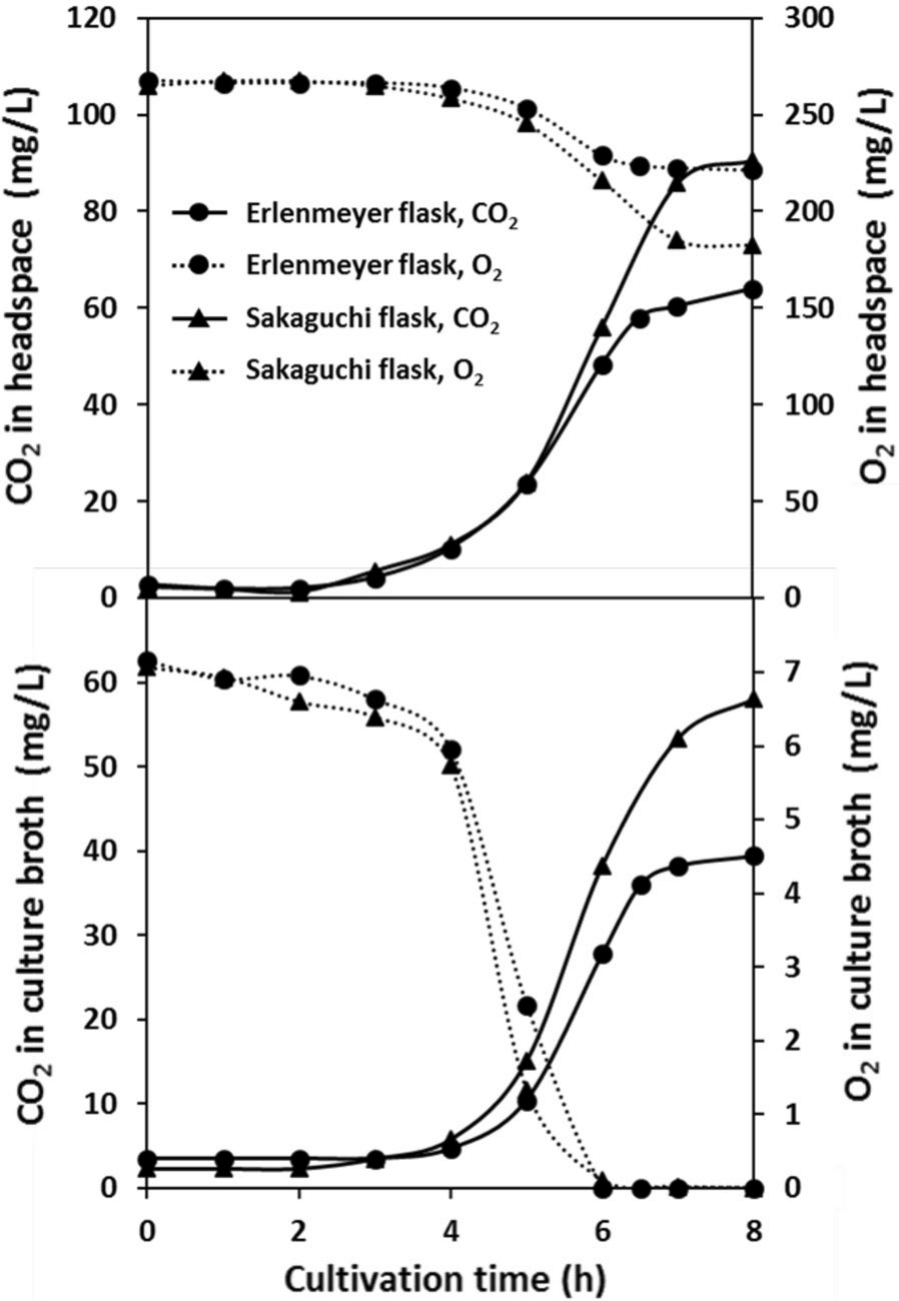
Real-time CO_2_ and O_2_ concentrations in conventional flasks in shake culture of *E. coli* K12 IFO3301. Shake-flask culture conditions for both flasks were set as 100 mL of LB medium at 30 °C, and 200 rpm in the case of Erlenmeyer flask and 120 strokes/min shaking frequency and 70 mm shaking diameter in the case of Sakaguchi flask. Both 500-mL Erlenmeyer and Sakaguchi flasks were equipped with breathable culture stoppers and CDMSS

In our previous study, the concentrations of CO_2_ and O_2_ in the gas–liquid phases were monitored using CDMSS in shake culture (Takahashi et al. 2017). In this study, CDMSS was used to monitor the headspace and the dissolved gases in various shake-culture flasks. The results are expressed in mean in Figs. 1 and 4, and were confirmed to be highly reproducible as in the case of development of CDMSS (Takahashi et al. 2017). A gaseous gradient tends to form in the headspace of the flask when microorganisms present under rich nutrient sources. In this study, CO_2_ and O_2_ in the flask gas phase were monitored under well-mixed conditions by gaseous circulation using CDMSS. No sedimentation or clogging of the cells was observed in the circulation system of shake flask culture.

## Results

**Fig. 2:**
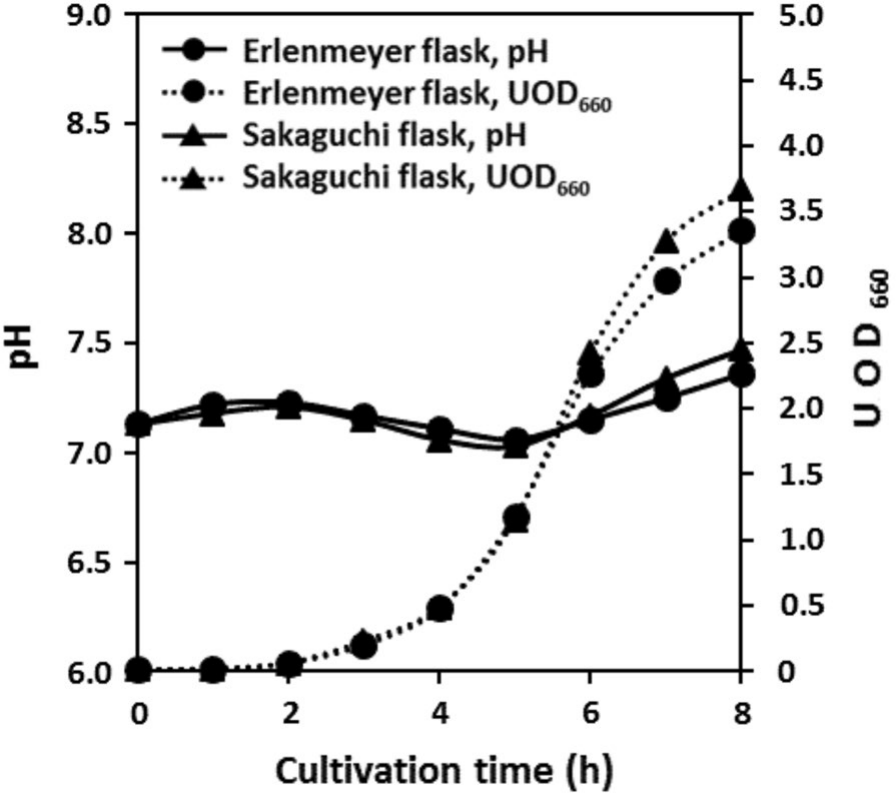
Changes in culture factors in the conventional shake-flask cultures

At the first, we compared typical shake flask cultures of *E. coli* K12 IFO3301 performed with Erlenmeyer and Sakaguchi flasks. *E. coli* K12 IFO3301 shake culture in Erlenmeyer and Sakaguchi flasks with CDMSS resulted in similar dissolved oxygen concentration (Fig. 1). *E. coli* K12 IFO3301 growth was gradual for 2 to 4 h and then exponential from 4 to 7 h (Fig. 2). The dissolved oxygen concentration gradually decreased till 4 h, then declined sharply and was depleted by 6 h (Fig. 1). Although the O_2_ concentration in the headspace was not depleted, it decreased in parallel with the decrease in dissolved oxygen concentration. The CO_2_ concentration in the headspace and culture broth increased as the O_2_ concentration decreased (Fig. 1). There was almost no significant difference in pH, cell growth, or dissolved oxygen concentration between the cultures in the Erlenmeyer and Sakaguchi flasks (Figs. 1 and 2). In Erlenmeyer and Sakaguchi flasks, *E. coli* K12 IFO3301 grew to some extent after the dissolved oxygen was depleted. Further, it plateaued, suggesting that O_2_ availability was rate limiting for growth (Figs. 1 and 2). Even though concentration of CO_2_ in the culture broth differed between the Erlenmeyer and Sakaguchi flasks, almost no difference in pH or growth was observed. In the conventional shake-flask culture, very large CO_2_ accumulations were observed in the gas and liquid phases compared to the atmosphere condition. The maximum CO_2_ concentration in culture broth and headspace was 40 mg/L and 63 mg/L in Erlenmeyer and 60 mg/L and 91 mg/L in Sakaguchi flasks, respectively (Fig. 1).

**Fig. 3:**
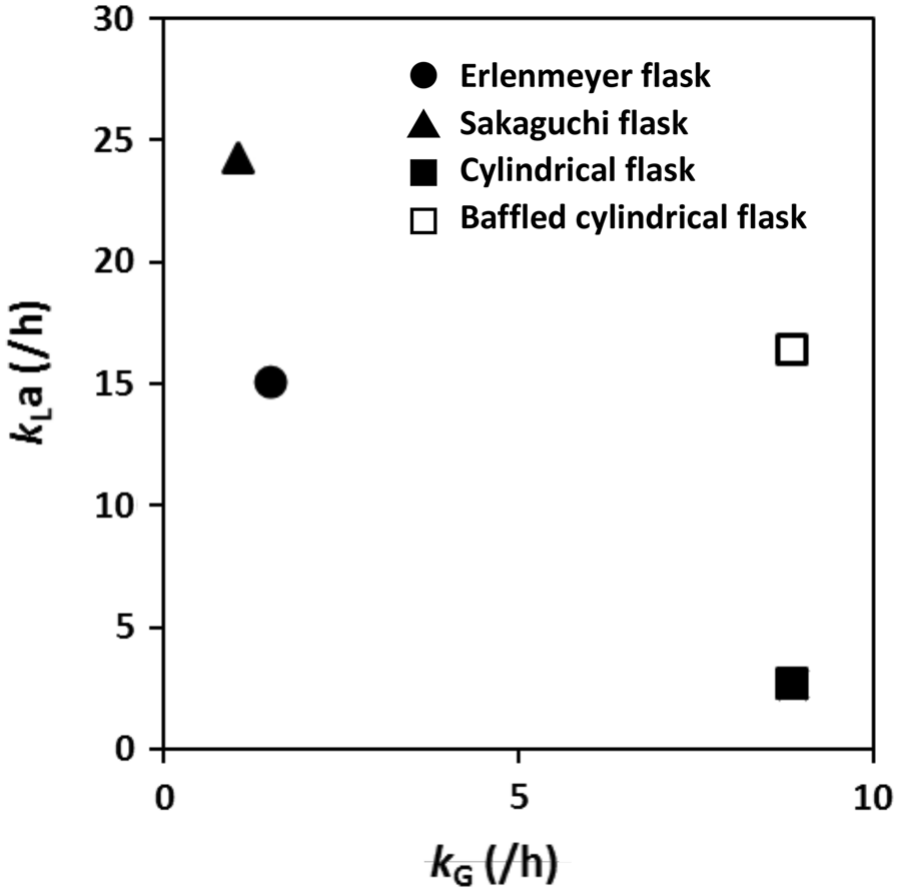
Relationship between *k*_L_a and *k*_G_ in various shake flasks

**Fig. 4:**
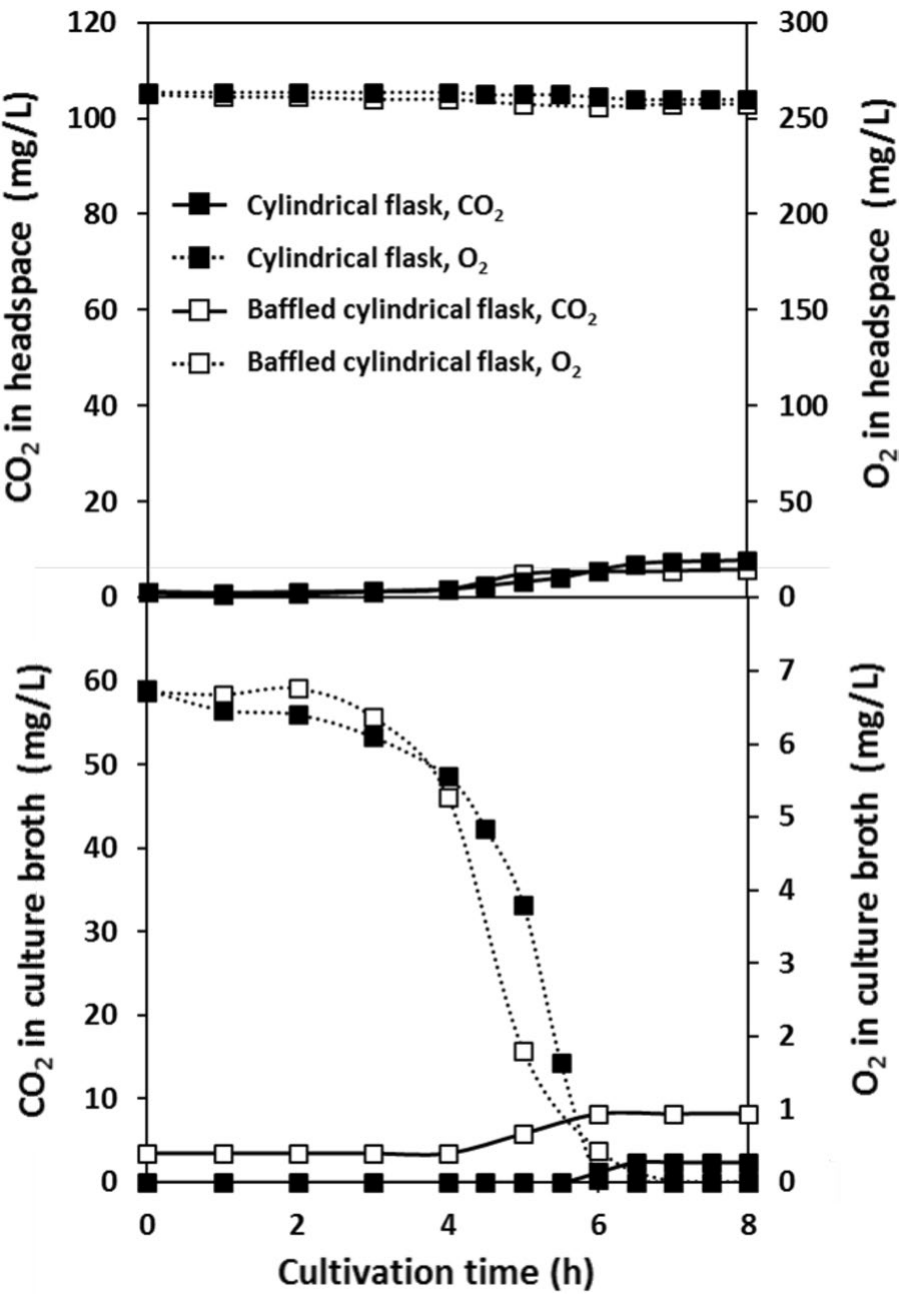
Real-time CO_2_ and O_2_ concentrations in cylindrical flasks during shaking culture of *E. coli* K12 IFO3301. Shake-flask culture conditions were 100 and 50 mL of LB medium at 30 °C, 200 rpm with 70 mm shaking diameter, and 500-mL cylindrical flask without baffle and 500-mL baffled cylindrical flask, both equipped with breathable culture stoppers and CDMSS

**Fig. 5:**
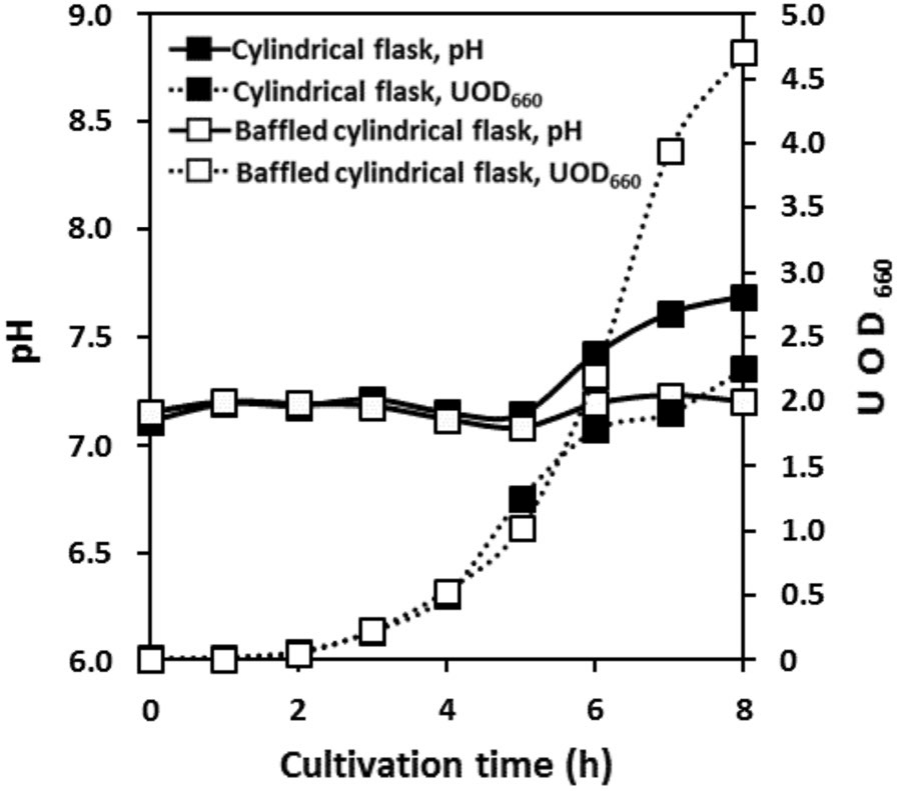
Changes in culture factors in shake culture using cylindrical flask and baffled cylindrical flask

Oxygen supply capacity and ventilation capacity were quantified for comparison in different flasks and shaking conditions. The *k*_L_*a* and *k*_G_ values measured in various shake-culture flasks with CDMSS are shown in Fig. 3. The time course of *E. coli* K12 IFO3301 shake culture is also presented in Figs. 4 and 5 by using cylindrical flask and baffled cylindrical flask. *E. coli* K12 IFO3301 grew at a similar rate in all the flasks for up to 5 h, at which point, growth reached a steady state in the cylindrical flask and increased in the other flasks (Figs. 2, 5). The measured maximum UOD_660_ decreased in the following order: baffled cylindrical flask = 4.70, Sakaguchi flask = 3.67, Erlenmeyer flask = 3.36, cylindrical flask = 2.25 (Figs. 2, 5). The dissolved oxygen concentration decreased similarly in all the flasks and was depleted after 6 h (Figs. 1, 4). The O_2_ concentration in the headspace of the Erlenmeyer and Sakaguchi flasks decreased, but it remained the same as its initial value in the cylindrical flask and baffled cylindrical flask. Cylindrical flask and baffled cylindrical flask maintained very low CO_2_ concentrations in the headspace and culture broth compared with Erlenmeyer and Sakaguchi flasks (Figs. 1, 4). The pH of the culture broth was the same in all the flasks during the first half of the shake culture but increased after 5 h in the cylindrical flask and decreased after 7 h in the baffled cylindrical flask, compared with those in the Erlenmeyer and Sakaguchi flasks (Fig. 5). In all the shake-culture flasks, the growth course of *E. coli* K12 IFO3301 cannot be explained only by *k*_L_*a*. For example, the Sakaguchi flask—with a low *k*_G_ and a high *k*_L_*a*—was slightly superior to the Erlenmeyer flask in terms of cell growth (Fig. 3), as the *k*_L_a of the Sakaguchi flask was 1.61 times that of the Erlenmeyer flask ([*k*_L_a of Sakaguchi flask: 24.3/*k*_L_a of Erlenmeyer flask: 15.1]) and the maximum growth in the Sakaguchi flask was 1.1 times that in the Erlenmeyer flask ([UOD_660_ of Sakaguchi flask: 3.67/UOD_660_ of Erlenmeyer flask: 3.36]). The maximum cell growth rate, *k*_G_, and *k*_L_a in baffled cylindrical flask were 2 times higher (4.7 in baffled cylindrical flask/2.3 in cylindrical flask), identical, and 5.9 times higher (16.4 in baffled cylindrical flask/2.8 in cylindrical flask) than those in the cylindrical flask, respectively.

## Discussion

We used Erlenmeyer (rotary shaker with 70 mm shaking diameter at 200 rpm) and Sakaguchi (reciprocating shaker with 70 mm shaking diameter at 120 strokes/min) flasks for shaking conditions of *E. coli* K12 IFO3301 cultivation. These shaking conditions are standard about batch culture on flask scale. It was suggested that the log-phase growth of *E. coli* K12 IFO3301 can be predicted by monitoring CO_2_ in the headspace of conventional shake flasks, because the CO_2_ concentration and the growth of *E*. *coli* K12 IFO3301 showed a very good correlation. Similar data have been reported by using a fluorescent sensor (Ge and Rao 2012) and CDMSS (Takahashi et al. 2017) in shaking culture by Erlenmeyer flask. These phenomena occur when the CO_2_ due to the respiratory activity of *E. coli* begins to fill the flask gas phase. However, in the cylindrical flask that could not supply enough oxygen, *E. coli* K12 IFO3301 did not grow much and the CO_2_ concentration remained almost unchanged. In the case of very low respiratory activity and need for detailed data, highly sensitive monitoring is required such as RAMOS.

We have an approach about shake-flask culture that goes beyond monitoring. It is possible that conventional shake culture using Erlenmeyer and Sakaguchi flasks has not previously taken into consideration the effect of CO_2_ accumulation in aerobic culture. We reported that *E. coli* growth improves in shake culture in Erlenmeyer and Sakaguchi flasks when the CO_2_ of flask gas phase, which accumulates due to respiratory activity, is maintained at low concentration by CDMSS with gaseous CO_2_ adsorbent (Takahashi et al. 2017; Takahashi and Aoyagi 2018c). This supports the idea that CO_2_ concentration in flask headspace has a significant impact on culture growth rate. This study compares different flasks by quantifying ventilation capacity and proves the above idea. Despite the almost same *k*_L_*a* in both flasks, the *k*_G_ ratio (Baffled cylindrical flask/Erlenmeyer flask) was 6.4 (8.9/1.4) and the maximum growth ratio (Baffled cylindrical flask/Erlenmeyer flask) was 1.4 (4.7/3.4). The CO_2_ concentration in baffled cylindrical flask and cylindrical flask was identical, despite the former supporting higher cell growth. This may be due to not only lower amount of medium and larger headspace but also sufficient ventilation in the baffled cylindrical flask. It is true that *E. coli* K12 IFO3301 growth requires a high *k*_L_*a*, but we conclude that not only high *k*_L_*a* but also high *k*_G_ is required by comparison of various flasks. It was noted that the detailed monitoring of flask gas phase in shaking culture became difficult in the case of the flask with high ventilation capacity.

CO_2_ has been reported to have a significant impact on culture growth (Blombach and Takors 2015, Takahashi and Aoyagi 2018a). Furthermore, CO_2_ concentration in the headspace can be maintained at low levels by adding adsorbent to the bypass part of CDMSS (Takahashi et al. 2017; Takahashi and Aoyagi 2018c). However, it may be difficult to set monitoring devices and adsorbent to multiple shake flasks when screening for secondary metabolites and developing early-stage bioprocesses for microorganisms (e.g., when examining culture conditions). In this study, we used baffled cylindrical flask, which has a headspace environment with a low CO_2_ accumulation without using CO_2_ adsorbent. However, the handling of flask set on the shaking table was the same as that of the conventional flask, but when the medium volume was 100 mL without baffled, the oxygen supply capacity was significantly reduced due to the lack of formation of thin water film on the flask wall. Hence, we ensured that *k*_L_a increased by inserting a baffle and lower working volume, and allowing the culture broth that climbed up the wall of the flask to hit the baffle during rotary. At that time, the working volume was also changed, but the oxygen supply capacity was quantified so that comparison was possible even under different flask conditions. Therefore, we expect that redesign of flasks based on not only *k*_L_a but also *k*_G_ (as shown in Fig. 3) may contribute to a wide range of fields employing cell culture.

## Abbreviations

CDMSS: Circulation direct monitoring and sampling system
RAMOS: Respiration Activity Monitoring System
UOD_660_: Unit optical density at 660 nm
